# Transcriptomic Characterization of Temperature Stress Responses in Larval Zebrafish

**DOI:** 10.1371/journal.pone.0037209

**Published:** 2012-05-30

**Authors:** Yong Long, Linchun Li, Qing Li, Xiaozhen He, Zongbin Cui

**Affiliations:** 1 The Key Laboratory of Aquatic Biodiversity and Conservation, Institute of Hydrobiology, Chinese Academy of Sciences, Wuhan, Hubei, People’s Republic of China; 2 Department of Biotechnology, Xiamen Ocean Vocational College, Xiamen, Fujian, People’s Republic of China; Temasek Life Sciences Laboratory, Singapore

## Abstract

Temperature influences nearly all biochemical, physiological and life history activities of fish, but the molecular mechanisms underlying the temperature acclimation remains largely unknown. Previous studies have identified many temperature-regulated genes in adult tissues; however, the transcriptional responses of fish larvae to temperature stress are not well understood. In this study, we characterized the transcriptional responses in larval zebrafish exposed to cold or heat stress using microarray analysis. In comparison with genes expressed in the control at 28°C, a total of 2680 genes were found to be affected in 96 hpf larvae exposed to cold (16°C) or heat (34°C) for 2 and 48****h and most of these genes were expressed in a temperature-specific and temporally regulated manner. Bioinformatic analysis identified multiple temperature-regulated biological processes and pathways. Biological processes overrepresented among the earliest genes induced by temperature stress include regulation of transcription, nucleosome assembly, chromatin organization and protein folding. However, processes such as RNA processing, cellular metal ion homeostasis and protein transport and were enriched in genes up-regulated under cold exposure for 48 h. Pathways such as mTOR signalling, p53 signalling and circadian rhythm were enriched among cold-induced genes, while adipocytokine signalling, protein export and arginine and praline metabolism were enriched among heat-induced genes. Although most of these biological processes and pathways were specifically regulated by cold or heat, common responses to both cold and heat stresses were also found. Thus, these findings provide new interesting clues for elucidation of mechanisms underlying the temperature acclimation in fish.

## Introduction

Environmental temperature variations affect many properties and functions of biomolecules and structural components of the cell, such as folding, assembly, activity and stability of proteins [1], structure and rigidity of lipids [2], [3], and fluidity and permeability of cell membrane [4], [5]. Due to the ubiquitous temperature dependence of structures and functions of various cellular elements, even small temperature changes would adversely disturb cellular homeostasis and attenuate physiological performance. The body temperature of most fishes equilibrates rapidly with ambient temperature, so water temperature is suggested to be the abiotic master factor which virtually controls and limits all the biochemical, physiological and life history activities [6], [7]. Under natural conditions, fishes may experience various sources of temperature fluctuations, including thermocline temperature variation, rapid changes in solar heat, abnormal water movements, rapid precipitation events or changes in seasonal temperatures [7]. To combat the adverse effects elicited by temperature fluctuations and maintain normal cellular functions at changed temperature, fishes have evolved versatile mechanisms that enable them to survive harsh environments with temperatures ranging from −2°C in the polar oceans to over 45°C in hot springs [8]. Some eurythermal fishes may adapt seasonally to temperatures from near freezing to over 36°C [9] and even endure a daily temperature cycle over a 20°C range [10].

The success of fishes in adaptation to an enormous range of environments has therefore led to considerable efforts on investigating how fishes respond and acclimate to temperature disturbance. Results from early physiological and biochemical studies have indicated that fishes may adapt to temperature variation through “biochemical restructuring”, namely changing the quantities of certain molecular species and the types of molecules present in the cells [11]. Many aspects of cellular biochemistry are involved in this restructuring process and well-defined adaptive responses include producing temperature specific isozymes [11], [12], changing the content of membrane lipid and the degree of fatty acid unsaturation [13], recruiting different muscle fiber types [14], synthesizing molecular chaperones [15], [16], changing mitochondrial densities and their properties [17], [18], and generating antifreeze protein during long-term evolutionary adaptation to freezing environment [19]. Obviously, these findings have elucidated the biochemical basis of adaptive responses to temperature stresses in fish.

It is well accpted that the biochemical changes induced by temperature stress are attributed to modulations of gene expression [14], [20]. In the last decade, microarray techniques have fundamentally revolutionized the investigations of gene regulation in organisms exposed to environmental challenges. Researchers have characterized the transcriptional responses elicited by hypo- or hyperthermia stress in fishes including common carp (*Cyprinus carpio*) [21], zebrafish (*Danio rerio*) [22], [23], channel catfish (*Ictalurus punctatus*) [9], annual killifish (*Austrofundulus limnaeus*) [10], coral reef fish (*Pomacentrus moluccensis*) [24], [25], goby (*Gillichthys mirabilis*) [26], [27], and rainbow trout (*Oncorhynchus mykiss*) [28]. A large number of genes were found to be regulated by temperature stress. In species like rainbow trout, channel catfish and common carp, differently expressed genes after exposure to temperature stress constitute about 10 percent of the investigated genes and most of which are upregulated. The upregulated genes are known to participate in a wide range of biological processes, such as transcriptional regulation, signal transduction, cell growth and differentiation, protein synthesis, stress response and metabolism regulation. Therefore, the transition to cold- or heat-acclimated state involves extensive changes in gene expression.

Although results from previous studies significantly advanced our understanding in the biochemical and molecular mechanisms underlying the adaptive response of fishes to temperature stress, several basic questions remain to be addressed. First, all of the previous studies have focused on the transcriptional regulation in adult tissues including brain, heart, liver, gill, kidney, skeletal muscle and intestine. Fish at larval stage fish are reported to be more susceptible to temperature variation [29], so it is not clear whether larva-specific responses to temperature stresses exist. Second, most previous studies were conducted with non-model species. The limited genetic resources severely restricted the number of genes for investigation and interpretation of the expression profiles.

Zebrafish are now widely used as a model animal for a variety of biological disciplines. Substantial information and resources for genetics, developmental biology, biochemistry, physiology and temporal-spatial gene expression patterns make zebrafish an ideal model for environmental genomics researches. Zebrafish is a small eurythermal fish that naturally lives in shallow fresh-water habitats and can withstand a wide range of daily and seasonal temperature fluctuations [30], [31]. It is likely that an efficient mechanism has evolved in zebrafish to cope with rapid changes in body temperature. In this study, we aimed to identify variations in gene transcriptional expression of zebrafish larvae during their acclimation to temperature changes. The Agilent Zebrafish Oligo Microarray (V2) (4×44K) was utilized to investigate the transcriptional responses of zebrafish larvae to cold (16°C) or heat (34°C) stress. This microarray platform contains 43603 synthesized oligonucleotide probes corresponding to at least 21794 unigenes. The combination of a well-established model organism with a microarray platform for a large number of genes would provide more detailed and accurate insights into molecular mechanisms underlying the adaptive response of fish to thermal stress. In addition, a parallel comparison of stress responses to temperature changes in different directions may identify cold- and heat-specific genes and signaling pathways in fish.

## Results

### Experimental Design and Effects of Temperature Stress on the Development of Zebrafish Larvae

**Figure 1:**
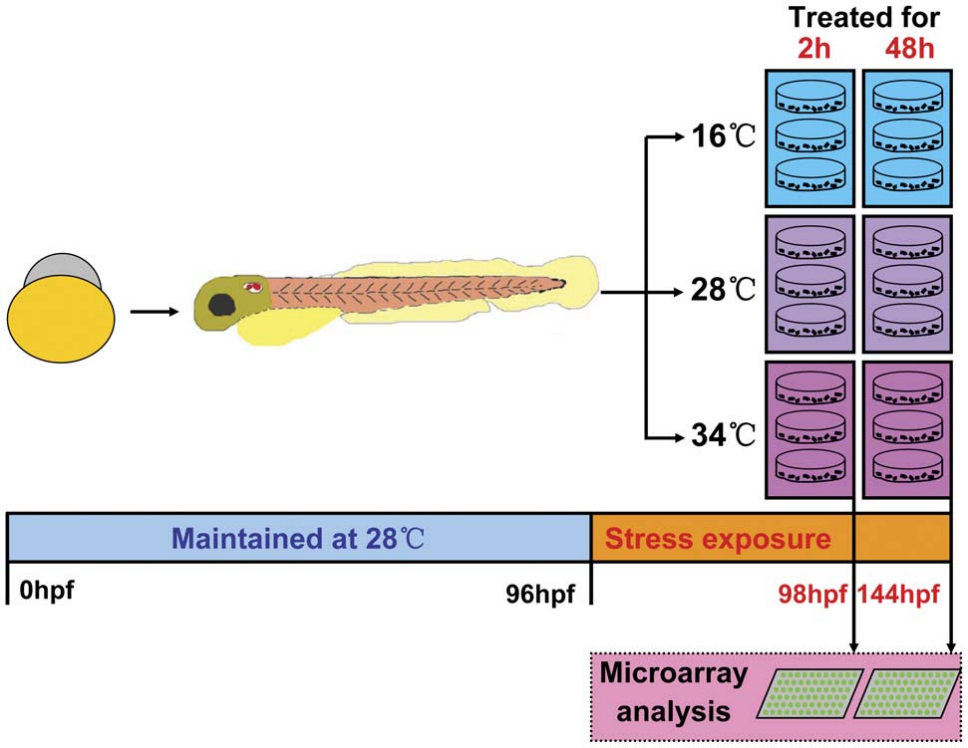
Experimental design. Zebrafish embryos were maintained at 28°C from fertilization to 96 hpf. Larvae at 96 hpf were exposed to 16, 28 or 34°C for 2 and 48 h, respectively. Samples exposed to different temperature were collected at 98 and 144 hpf and subjected to microarray analysis.

To characterize the transcriptional responses of genes regulated by cold or heat stress in larval zebrafish, larvae at 96 hpf (maintained at 28°C from fertilization, designated as 28°C-96 hpf) were exposed to 16 (cold), 28 (control) or 34°C (heat) for 2 (from 96 to 98 hpf) and 48 h (from 96 to 144 hpf), respectively. The expression of genes in samples of these six groups (designated as 16°C-2 h, 16°C-48 h, 28°C-98 hpf, 28°C-144 hpf, 34°C-2 h and 34°C-48 h, respectively) was analyzed using Agilent microarray (Figure 1). This experimental design was aimed to reveal both immediate and later transcriptional events regulated by temperature stress.

**Figure 2:**
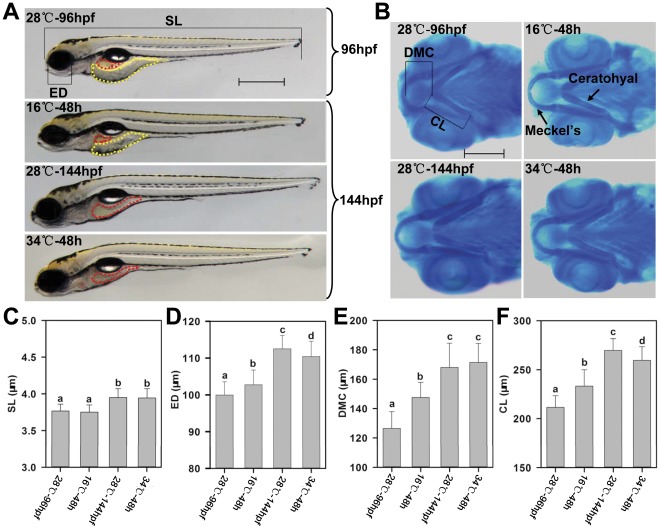
Effects of temperature stress on the development of zebrafish larvae. (**A**) Representative images of zebrafish larvae before and after temperature stress. Zebrafish larvae at 96 hpf (maintained at 28°C from fertilization) were exposed to temperature stress for 48 h (cultured at 16, 28 or 34°C from 96 to 144 hpf). Images were taken under a stereomicroscope from Zeiss with a color CCD camera. Red and yellow dashed line indicates intestine lumen and yolk sac, respectively. SL: standard length (distance from the snout to the posterior tip of the notochord). ED: eye diameter. Scale bar: 500 µm. (**B**) Alcian blue staining of the cartilages of jaw and branchial arches. DMC: distance from the inner border of Meckel’s cartilage to the anterior end of ceratohyal. CL: ceratohyal length. Scale bar: 200 µm. (**C, D, E** and **F**) Bar charts demonstrate the effect of temperature stress on the SL, ED, DMC and CL, respectively. Horizontal axis indicates treatments described in (**A**) and (**B**). Error bars indicate standard deviations (n = 40–60). Different letters above the error bars indicate statistically significant differences determined by one-way analysis of variance (ANOVA) followed by Duncan’s multiple range test (*p*<0.05).

After exposure to 16 or 34°C for 48 h (from 96 to 144 hpf), the morphology of treated larvae (16°C-48 h and 34°C-48 h) were compared with that of corresponding controls (28°C-144 hpf) to determine the effects of temperature stress on the development. As shown in Figure 2A, the 16°C -48 h larvae displayed phenotypes more like those of 28°C-96 hpf larvae and demonstrated a larger yolk sac and smaller intestine lumen in comparison with those of control larvae. However, no obvious difference in morphology was observed between 34°C-48 h and 28°C-144 hpf larvae (Figure 2A). The standard length (SL) and eye diameter (ED) of larvae from different groups were measured and compared to represent the process of development. As shown in Figure 2C, the SL of 16°C-48 h larvae is significantly shorter than those of 28°C-144 hpf and 34°C-48 h larvae. Similarly, the ED of 16°C -48 h larvae is smaller than those of larvae in other two groups, but it is larger than that of 28°C -96 hpf larvae (Figure 2D). Heat stress markedly inhibited the increase of ED, but this effect is lower than that of cold stress (Figure 2D).

To address whether temperature stress influences the growth of body length and eye diameter but not the development of other organs, alcian blue staining was performed to determine the cartilage development of jaw and branchial arches (Figure 2B) by measuring the distance from the inner border of Meckel’s cartilage to the anterior end of ceratohyal (DMC) and ceratohyal length (CL). These parameters have previously been used to evaluate the cartilage development of zebrafish larvae [32]. The results of statistical analysis indicate that the DMC and CL of 16°C-48 h larvae are significantly smaller than those of 28°C-144 hpf larvae (Figure 2E and F). Heat stress inhibited the increase of CL as well (Figure 2F); however, the DMC and CL values of larvae exposed to both cold and heat are significantly larger than those of larvae at the beginning of exposure (28°C-96 hpf).

Taken together, these data suggest that the development of zebrafish larvae was not stopped, but significantly delayed under cold stress. The inhibitory effect of heat stress on the development of zebrafish larvae is lower than that of cold stress.

### Effects of Temperature Stress on the Body Composition

The main body composition parameters of zebrafish larvae were analyzed to elucidate the effects of temperature stress on the nutrient consumption and energy conversion. As shown in Table 1, the wet mass of zebrafish larvae was not significantly affected by temperature stress exposure for 48 h; however, the dry mass, protein, lipid and glycogen content of 16°C-48 h larvae were all higher than those of 28°C-144 hpf larvae. It is interesting that the glycogen content of 16°C-48 h samples was found to be higher than that of 28°C-96 hpf larvae. In comparison with 28°C-144 hpf samples, exposure to heat stress for 48 h significantly reduced the dry mass, protein and glycogen contents of zebrafish larvae. These results suggest that cold and heat stress exert opposite effects on the nutrient consumption in developing larvae, and this is consistent with the delayed development under cold stress.

**Table 1:** Body composition of zebrafish larvae before and after temperature stress exposure.

Components	28°C-96 hpf	16°C-48 h	28°C-144 hpf	34°C-48 h
Wet mass (n = 5)	367.67±13.87^a^	413.67±22.68^b^	421.33±10.83^b^	398.33±22.58^b^
Dry mass (n = 5)	62.67±0.91^a^	59.67±2.17^b^	56.33±0.75^c^	50.33±3.21^d^
Protein (n = 10)	55.37±4.74^a^	55.72±3.13^a^	49.98±3.96^b^	45.07±3.82^c^
Lipid (n = 5)	10.40±0.80^a^	10.12±0.40^a^	7.82±0.62^b^	7.87±0.37^b^
Glycogen (n = 5)	0.48±0.03^a^	0.56±0.05^b^	0.33±0.07^c^	0.21±0.04^d^

Notes: 1) Zebrafish larvae at 96 hpf were exposed to 16, 28 or 34°C for 48 h. Samples exposed to different treatments were designated as 28°C -96 hpf, 16°C -48 h, 28°C -144 hpf and 34°C -48 h, respectively. 2) Data (µg/individual) are given as means ± standard deviations. The number of samples is displayed in parenthesis. 3) The numbers of individuals used for the measurement of wet mass, dry mass, protein, lipid and glycogen are 80, 80, 1, 20 and 20 respectively. 4) Different letters in the same row indicate statistically significant differences determined by ANOVA followed by Duncan’s multiple range test or Dunnett’s T3 test (*p*<0.05).

### Global Effects of Temperature Stress on Gene Expression

**Figure 3:**
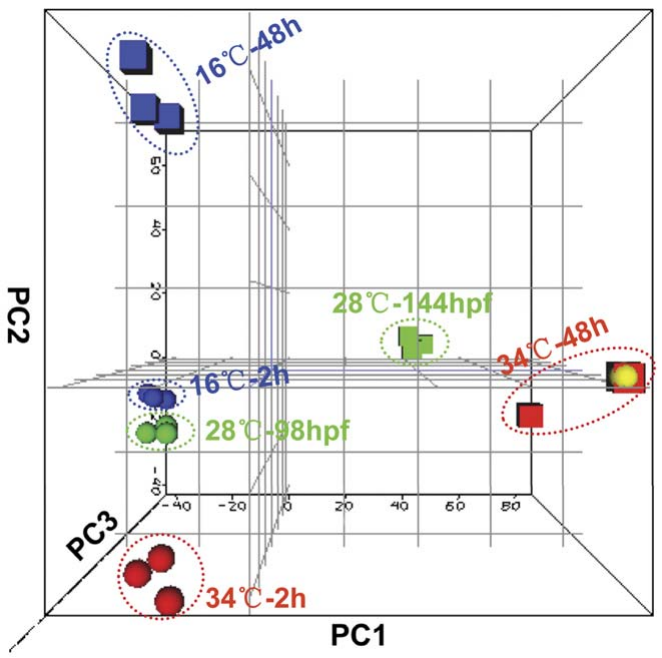
Principle component analysis (PCA) of gene expression profiles. Normalized signal intensity values of all the probes called “present” in at least 2/3 (12) arrays were subjected to PCA using ArrayTrack. The x-, y- and z-axes represent PC1, PC2 and PC3, respectively. The colors and shapes of data points indicate temperature treatment and time of exposure: blue for cold (16°C), red for heat (34°C) and green for control (28°C), sphere for 2 h and square for 48 h after exposure. One of two overlapped heat-treated samples at 48 h is shown as yellow and sphere. The sample names were displayed within the figure.

To examine overall effects of cold or heat stress on gene transcription, signal intensity values of all probes with significant expression were subjected to principle component analysis (PCA). The results of PCA indicate that 72.5% of the transcriptional variations can be explained by the first three components. As shown in Figure 3, the projections of all samples in the principle component space clearly revealed an obvious consistency within the same group and a clear discrepancy among groups. The first principle component (PC1) mainly captured the difference between 98 and 144 hpf larvae maintained at 28°C (Figure 3), indicating the expression variations of genes associated with the normal development process. A similar trend of variation in gene expression between larvae exposed to heat stress for 2 and 48 h was also explained by PC1 (Figure 3). PC2 mainly captured the difference in gene expression between cold-treated and control samples (Figure 3). PC3 mainly explained the variation in gene expression between larvae maintained at 28°C and 34°C (Figure 3).

### Validation of Microarray Data by Quantitative Real-time PCR (qPCR)

To validate the expression profiles from microarray analysis, relative mRNA levels for 15 genes were measured by qPCR. To choose the most stable genes as internal references for qPCR data normalization, five candidates were selected according to their expression levels detected by microarray. The expression of these five genes was also measured by qPCR. The software NormFinder [33] was used to calculate the intra- and inter-group variations in their expression. The results indicate that *tnnt3b* (troponin T3b, skeletal, fast) is the most stable gene, whereas *tmpa* (alpha-tropomyosin) and *tnnt3b* are the best two-gene combination (Table S2). Thus, the geometric mean of Cq values and primer efficiency values of these two genes were used for normalization.

**Figure 4:**
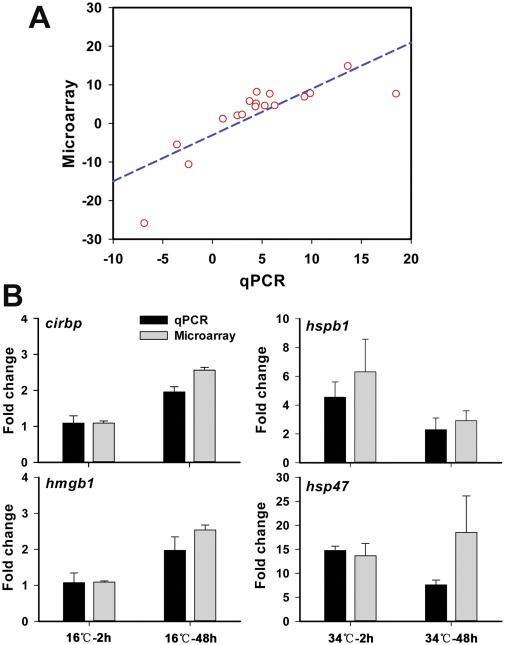
Validation of microarray data by qPCR. (**A**) Correlation between qPCR (x-axis) and microarray (y-axis) data. The numeric expression values of genes in selected samples were displayed in Table S3. The correlation between microarray and qPCR data were analyzed by Spearman’s rho test. Highly statistical significance [r (17) = 0.916, p = 0.00001] was observed. (**B**) Pair wise comparison of microarray and qPCR data for temperature stress-related marker genes. The expression of *cirbp* and *hmgb1* was detected in cold-treated samples (16°C-2 h and 16°C-48 h), and the expression of *hspb1* and *hsp47* were detected in heat-treated samples (34°C-2 h and 34°C-48 h), respectively. Error bars indicate standard deviations (n  = 3).

The expression data for 15 selected genes detected by microarray and qPCR are listed in Table S3 and plotted in Figure 4A. The qPCR and microarray methods showed excellent qualitative agreement on both up- and down-regulated genes. The correlation between microarray and qPCR data was analyzed by Spearman’s rho test and a highly statistical significance [r (17) = 0.916, p = 0.00001] was observed. The expression of several cold- or heat-specific genes identified in previous studies was also analyzed to evaluate the data reliability and the sensitivity of microarray analysis. As shown in Figure 4B, the induced expression of cold marker genes *cirbp* (cold inducible RNA binding protein) and *hmgb1* (high-mobility group box 1) [21], [23] and heat marker genes *hspb1* (heat shock protein, alpha-crystallin-related, 1) and *hsp47* (heat shock protein 47) [27] was successfully detected by both qPCR and microarray approaches. These results confirmed the reliability of the single-channel microarray analysis method in this study.

### Differentially Expressed Genes Under Temperature Stress and During Developmental Processes

**Figure 5:**
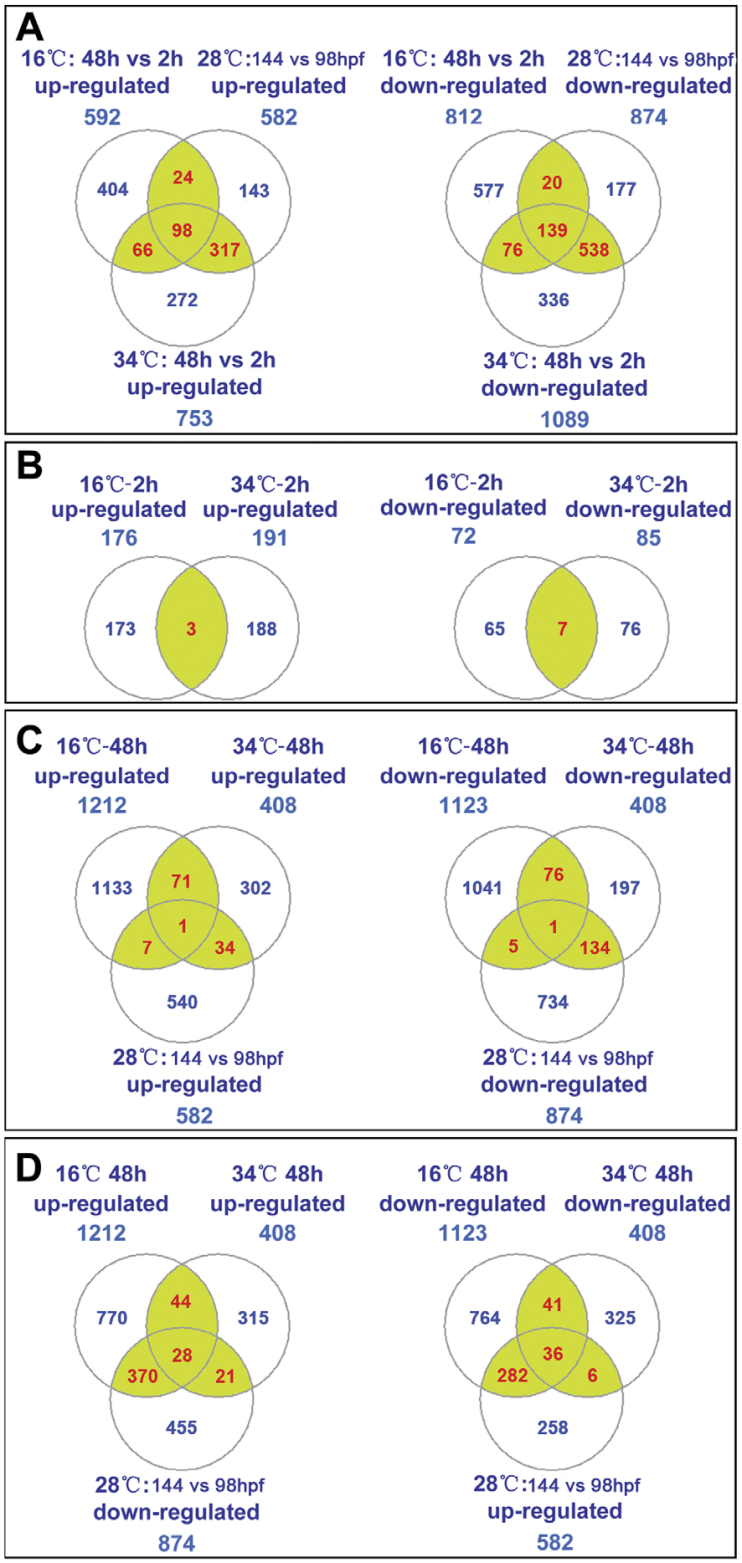
Venn diagrams represent the number of differentially expressed genes. (A) Developmental processes-associated genes under 16, 28 and 34°C. Differentially expressed genes were identified by comparing their expression in 144 hpf samples (48 h after exposure) to that in 98 hpf samples (2 h after exposure) at the same temperature. (**B**) Temperature stress-regulated genes after 2 h of exposure. Differentially expressed genes were identified by comparing the expression of genes in 16°C-2 h and 34°C-2 h samples to that in 28°C-98 hpf samples. (**C** and **D**) Overlap between temperature-regulated genes and developmental processes-associated genes. Genes differentially expressed in 16°C-48 h and 34°C-48 h samples were compared to the development processes-associated genes identified from larvae maintained at 28°C?(28°C-144 hpf vs. 28°C-98 hpf). The official symbols of genes differentially expressed under different situations were displayed in Table S6.

The expression of developmental processes-associated genes under different temperatures was displayed in Table S4. The total and overlapping numbers of up- and down-regulated genes among groups were shown in Figure 5A. Differential expression of genes in larvae at 28°C are mainly ascribed to the normal developmental process, while expression variations of genes in larvae exposed to temperature stress are attributed to both stress response and developmental processes. After treatment for 48 h, larvae exposed to heat stress led to more differentially expressed genes than those exposed to cold stress (Figure 5A). In addition, 55% (415/753) of up-regulated genes and 62% (677/1089) of down-regulated genes in heat-treated larvae were overlapped with those associated with normal developmental processes; however, the numbers in cold-treated larvae were 21% (122/592) and 20% (159/812), respectively.

The expression of temperature-regulated genes was displayed in Table S5. The numbers of genes regulated by cold or heat stress after exposure for 2 and 48 h are shown in Figure 5B and 5C, and a number of genes were found to be regulated by both cold and heat stress. In comparison with those at 2 h, the numbers of both up- and down-regulated genes at 48 h were markedly increased. To clarify whether these genes are specifically regulated by temperature, the overlapping numbers of temperature-regulated and developmental processes-associated genes were calculated. As shown in Figure 5C, the portion of genes shared by cold stress and developmental processes was less than 1% of total identified genes (8/1212 and 6/1123 for up- and down-regulated genes, respectively). However, 9% (35/408) of up-regulated and 33% (135/408) of down-regulated genes under heat stress were associated with developmental processes.

Interestingly, 33% (398/1212) of cold-induced genes were down-regulated and 28% (318/1123) of cold-inhibited genes were up-regulated during the normal development process of larvae from 98 to 144 hpf (Figure 5D). Rank–rank Hypergeometric Overlap (RRHO) analysis [34] revealed a significant overlap between the reversed list of cold-regulated genes (16°C-48 h vs. 28°C-144 hpf) and the list of development processes-associated genes (28°C-144 hpf vs. 28°C-98 hpf) (Figure S2) and the significantly overlapped genes were displayed in Table S6. The differential expression of these genes is most likely resulted from developmental delay under cold stress but not related to the cold acclimation processes, so they were excluded from subsequent analyses.

### Gene Ontology (GO) Enrichment Analysis for Genes Regulated by Temperature Stress

**Figure 6:**
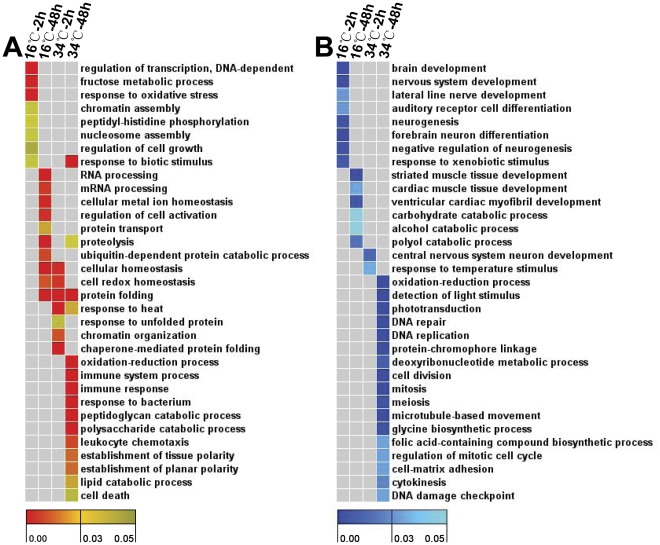
Heat maps of GO enrichment analysis for temperature-regulated genes. (**A**) Up-regulated genes. (**B**) Down-regulated genes. Genes up or down-regulated at each time point were submitted to GO enrichment analysis using GOEAST web-based software (http://omicslab.genetics.ac.cn/GOEAST/index.php). The results of GO enrichment analysis are displayed in Table S7. GO terms were selected to display in heat maps according to their statistical significance and locations in the GO tree. Columns and rows in the heat maps indicate treatments and enriched biological process GO terms, respectively. Sample names were displayed above the heat maps. Color scales indicate p values of enrichment tests and gray cells represent an empty value or a value >0.05.

To find out temporal transcriptional events occurred during the process of thermal acclimation, genes up- or down-regulated at each time point were subjected to GO enrichment analysis. The results of GO analysis were displayed in Table S7 and representative biological process terms were displayed in Figure 6. Overrepresented biological processes of genes specifically induced by cold after 2 h of exposure include regulation of transcription, peptidyl-histidine phosphorylation, nucleosome assembly and fructose metabolic process (Figure 6A). Transcriptional regulation was the most enriched term for genes immediately induced by cold stress (Figure S3) and more than 10% (23/176) of genes up-regulated at 2 h were annotated as transcription factors. Processes enriched in cold-induced genes after 48 h of exposure include RNA processing, cellular metal ion homeostasis, regulation of cell activation, protein transport and ubiquitin-dependent protein catabolic process (Figure 6A). Additionally, biological processes such as proteolysis, cellular homeostasis, protein folding and cell redox homeostasis, were enriched among both cold and heat stress induced genes (Figure 6A). Unlike the situation of cold stress, the most significant terms overrepresented among genes immediately up-regulated by heat exposure was chaperone-mediated protein folding (Figure S4), suggesting a significant difference between cold and heat acclimation.

GO analysis of the earliest genes specifically down-regulated by cold indicated that biological processes such as neurogenesis, brain development and response to xenbiotic stimulus were overrepresented (Figure 6B). At later time points, biological processes overrepresented among cold-inhibited genes include striated muscle structure development, carbohydrate catabolic process, alcohol catabolic process and polyol catabolic process (Figure 6B). Phototransduction, DNA repair, DNA replication and cell division were found to be enriched among genes specifically inhibited by heat stress (Figure 6B).

### Pathways Enriched in Temperature Stress-regulated Genes

**Figure 7:**
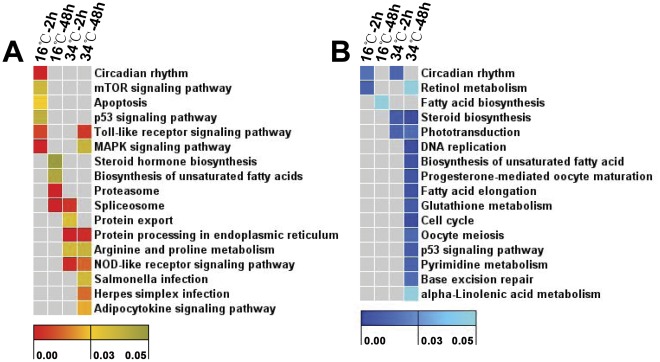
Heat maps of pathway enrichment analysis for temperature-regulated genes. (**A**) Up-regulated genes. (**B**) Down-regulated genes. Genes up or down-regulated by temperature stress at each time point were submitted to pathway enrichment analysis based on KEGG by ClueGO plugin of Cytoscape. Columns and rows in heat maps indicate treatments and enriched pathway terms, respectively. Sample names were displayed above the heat maps. Color scales represent *p* values of enrichment tests and gray cells indicate an empty value or a value >0.05.

Pathways including spliceosome, MAPK signaling and toll-like receptor signaling were enriched among cold- and heat-activated genes. The earliest pathways overrepresented in genes specifically induced by cold include circadian rhythm, apoptosis, mTOR signaling and p53 signaling (Figure 7A). These pathways are mainly involved in the regulation of biological processes such as immune reaction, environmental adaptation, signal transduction, and cell growth and death. Heat-specific pathways included protein export, protein processing in endoplasmic reticulum, adipocytokine signaling pathway and arginine and praline metabolism (Figure 7A).

In addition, nearly equal numbers of pathways were enriched in genes inhibited by temperature stress. Some pathways, such as retinol metabolism and circadian rhythm were overrepresented by both cold and heat-inhibited genes (Figure 7B). Only fatty acid biosynthesis was enriched among genes specifically inhibited by cold stress (Figure 7B). The most significant pathways overrepresented in heat-inhibited genes include steroid biosynthesis, biosynthesis of unsaturated fatty acids, DNA replication, fatty acid elongation, glutathione metabolism and cell cycle (Figure 7B). Some cold-specific pathways such as biosynthesis of unsaturated fatty acids and p53 signaling were found to be overrepresented by heat-inhibited genes (Figure 7A and B), indicating that cold and heat stresses may exert opposite effects on these biological processes. However, certain enriched pathways or GO terms may not reflect the real situations in some tissues or cells due to the use of whole organisms.

### Representative Genes Regulated by Temperature Stress

**Figure 8:**
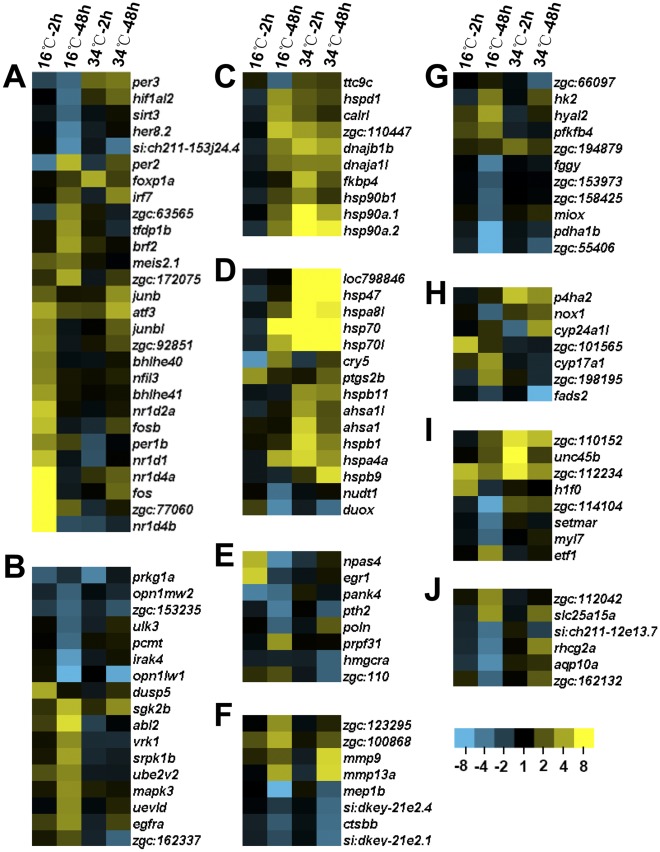
Expression profiles of genes in selected GO categories. (**A**) Regulation of gene expression**;** (**B**) Protein modification process; (**C**) Protein folding; (**D**) Response to stress; (**E**) Nucleic acid metabolic process; (**F**) Proteolysis; (**G**) Carbohydrate metabolic process; (**H**) Oxidation-reduction process; (**I**) Cellular component organization; (**J**) Transmembrane transport. All of the genes regulated by temperature stress were submitted to functional annotation using DAVID software [63] (http://david.abcc.ncifcrf.gov/tools.jsp) and 10 representative biological process GO categories were selected. Genes exhibiting a fold change ≥3.0 in at least one sample were displayed. Columns and rows in the heat maps represent samples and genes, respectively. Sample names were displayed above the heat maps. Color scale indicates fold changes of gene expression. Genes that belong to more than one functional category were displayed only once.

In this study, a total of 2680 genes were found to be specifically regulated by temperature stress (with a fold change ≥1.8, FDR <0.05, Table S5) and many of them are not identified as temperature-regulated genes in previous studies. To give an explicit picture about the expression of representative genes, genes exhibited a fold change ≥3.0 and involved in the 10 representative biological processes such as regulation of transcription, protein modification, protein folding and proteolysis, were displayed in Figure 8.

Transcription factors such as *fos* (v-fos FBJ murine osteosarcoma viral oncogene homolog), *zgc:77060* (or *nfil3–6*, nuclear factor, interleukin 3 regulated, member 6), *bhlhe41* (basic helix-loop-helix family, member e41), *bhlhe40* (basic helix-loop-helix family, member e40) and several members of the nuclear receptor subfamily 1, group d, such as *nr1d4b*, *nr1d4a*, *nr1d1* and *nr1d2a* were immediately induced after cold exposure (Figure 8A). Genes such as *per2* (period homolog 2 (Drosophila)), *zgc:63565* (or *rab11al*, RAB11a, member RAS oncogene family, like), *tfdp1b* (transcription factor Dp-1, b) and *brf2* (BRF2, subunit of RNA polymerase III transcription initiation factor, BRF1-like) were induced by exposure to cold stress for 48 h. Cold exposure also inhibited the expression of transcription factors such as *sirt3* (sirtuin (silent mating type information regulation 2 homolog) 3 (S. cerevisiae)), *hif1al2* (hypoxia-inducible factor 1, alpha subunit, like 2) and *her8.2* (hairy-related 8.2) (Figure 8A).

Genes involved in protein modification, such as *dusp5* (dual specificity phosphatase 5), *abl2* (tyrosine-protein kinase ABL2) and *srpk1b* (serine/arginine-rich protein specific kinase 1b) were the most prominent genes highly induced by cold (Figure 8B); *sgk2b* (serum/glucocorticoid regulated kinase 2b) was induced by both cold and heat. Among the cold-inhibited genes involved in protein modification, *zgc:153235* (or ppm1lb, protein phosphatase, Mg2+/Mn2+ dependent, 1Lb), *opn1lw1* (red-sensitive opsin-1) and *opn1mw2* (red-sensitive opsin-2) were also down-regulated by heat. *Opn1lw1* and *opn1mw2* are two duplicated red-sensitive opsin genes functioning as color sensors in fish [35].

Protein folding was overrepresented among both cold- and heat-induced genes (Figure 6A). Large heat shock protein genes including *hsp90a.1* (heat shock protein 90-alpha 1), *hsp90a.2* (heat shock protein 90-alpha 2) and *hsp90b1* (heat shock protein 90, beta (grp94), member 1) were induced by both cold and heat exposure (Figure 8C). Hsp90 proteins are unique molecular chaperones; they do not fold newly synthesized proteins [36]. It has been shown that these proteins play important roles in cellular signal transduction networks because the majority of their substrates are signal transduction proteins, such as steroid hormone receptors and signaling kinases [36]. Most of these genes involved in protein folding were highly up-regulated by heat stress immediately after exposure; however, cold exposure resulted in a lagged effect on the expression these genes.

Moreover, some genes involved in other biological processes were also affected by temperature stress. The expression of *hspa8l* (heat shock protein 8-like), *hsp70* (heat shock cognate 70-kd protein) and *hsp70l* (heat shock cognate 70-kd protein, like) were highly induced by heat stress immediately after exposure; however, their expression increased gradually in cold exposed larvae (Figure 8D). The expression of *hspb1* (heat shock protein, alpha-crystallin-related, 1) was specifically induced by heat exposure (Figure 8D) and this gene plays important protective roles in preventing protein misfolding and aggregation [37].

RNA processing gene *prpf31* (PRP31 pre-mRNA processing factor 31 homolog) were specifically induced by cold (Figure 8E). Mutation of *PRP31* in yeast (*Saccharomyces cerevisiae*) was reported to cause defects in processing of pre-RNA species and sensitivity to high temperature (37°C) [38]. However, up-regulation of *prpf31* was found in cold-exposed zebrafish larvae. Although most of the highly regulated genes involved in proteolysis were down-regulated by cold stress, *zgc:100868* and *mmp13a* (matrix metalloproteinase 13a), annotated as serine-type endopeptidase and metalloendopeptidase gene, were up-regulated (Figure 8F). Similarly, genes involved in carbohydrate metabolic processes including *pdha1b* (pyruvate dehydrogenase E1 alpha 1) and *fggy* (FGGY carbohydrate kinase domain containing) were down-regulated by cold exposure; however, genes involving *hyal2* (hyaluronoglucosaminidase 2) and *pfkfb4* (6-phosphofructo-2-kinase/fructose-2,6-biphosphatase 4) were highly up-regulated (Figure 8G). Representative gene involved in process including oxidation reduction (Figure 8H), cellular component organization (Figure 8I) and transmembrane transport (Figure 8J) were also shown.

## Discussion

Temperature represents one of abiotic factors essential for the development and growth of fish species. To investigate the regulation of fish genes by temperature stress at larvae stage, we characterized the transcriptional responses of zebrafish larvae to cold or heat stress using microarray analysis. The reliability and accuracy of microarray data were validated by qPCR. A large number of key biological processes, intracellular pathways and genes were identified to be involved in the process of temperature acclimation. In addition, a comparison of responses of zebrafish larvae to cold and heat stresses resulted in the discovery of many cold-specific biological events that are worth of further investigation. Therefore, the results provide key clues for elucidation of molecular mechanisms underlying the regulatory networks of gene expression during the temperature acclimation of fish and indicate that zebrafish larvae are suitable for deciphering undefined issues in environmental genomics.

Since developing zebrafish larvae at 96 hpf were utilized for temperature stress exposure, the effects of temperature stress on development was first determined. Due to the limitations of commonly accepted staging methods [39], [40] and relatively short exposure periods, morphological indices may not be enough for characterizing the effects of temperature stress on larvae development. It is previously shown that body size is a robust indicator of developmental progress and the high correlations between SL and developmental progress are relatively independent of rearing conditions [39]. Therefore, SL, ED, DMC and CL were used as parameters of the developmental progress in this study. The results indicate that the development of zebrafish larvae is not stopped, but significantly delayed under cold stress. Heat stress also exerts an inhibitory effect on the development of zebrafish larvae, but the extent is lower than that of cold stress.

The identification of important biological processes and master genes involved in the establishment of acclimated phenotypes after stress exposure remains a challenging issue in environmental genomics. Regulation of transcription, fructose metabolic process, peptidyl-histidine phosphorylation and nucleosome assembly are the most significant GO terms enriched in genes immediately up-regulated by cold stress; however, these biological process GO terms were not significantly enriched in genes up-regulated by heat stress, suggesting the specificity of these responses to cold stress. Some GO terms such as protein folding, oxidation-reduction process, cellular homeostasis and cellular component organization, were enriched in genes up-regulated by exposure to both cold and heat stress, indicating that common transcriptional responses can be elicited by temperature fluctuation in both directions. Thus, further investigations focusing on these biological processes could facilitate the identification of master factors controlling the intracellular signal transduction of temperature stress.

The up- and down-regulation of hundreds of genes under temperature stress occur in waves with the extension of incubation period and CBF transcription factors in Arabidopsis (*Arabidopsis thaliana*) are identified as major regulatory “hubs” to control the expression of a large number of genes [41]. In this study, more than 10% (23/176) of immediately cold-induced genes encode transcription factors. The candidate master genes include *fos*, *nr1d4a*, *nr1d4b*, *nr1d1*, *nr1d2a*, *bhlhe40* and *bhlhe41*. Among these genes, *nr1d4a*, *nr1d4b*, *nr1d1* and *nr1d2a* belong to the same subfamily (rev-erb) of nuclear receptors. Nuclear receptors are a class of proteins existing within cells that are responsible for the sense of small lipophilic molecules such as steroid and thyroid hormones, and products of lipid metabolism including fatty acids, prostaglandins, or cholesterol derivatives [42]. Both *nr1d1* (rev-erb alpha) and *nr1d2* (rev-erb beta) were reported to be transcriptional silencers involved in the regulation of circadian rhythm, lipid metabolism, and cellular differentiation [43], [44]. Thus, the up-regulation of these two genes may be associated with the repression of large number of genes after cold exposure. In addition, the induction of *nr1d1* and *nr1d2a* by cold stress and their involvement in circadian rhythm regulation suggest a relationship between temperature response and circadian clock regulation. Another potential central regulator in temperature acclimation is the Fos (C-Fos), a nuclear protein that can be induced by various stresses and acts to control the expression of many target genes [45]. The induction of *fos* by cold and heat stress has previously demonstrated in mammals [46], [47]. In consistence with the results from mammals, we have detected substantial induction of *fos* in zebrafish under both cold and heat stress. Therefore, the identification of target genes directly controlled by these transcription factors would reveal insights into molecular mechanisms underlying the regulation of temperature acclimation in fish.

Molecular mechanisms underlying the cold or heat resistance in animals are more complicated than that in plants, since animals possess a number of complex sensory organs and nervous systems. When exposed to ambient temperature fluctuations, mammals are able to keep their body temperature in a proper range through thermoregulation. It is well established that central nervous system (CNS) of mammals plays important roles in the thermoregulation. Among various brain structures, hypothalamus is suggested to be the most notable central thermoregulatory site [48], although other brain regions are also involved in thermal regulation [45]. The primary response to cold stress in fish is suggested to be a neuroendocrine response that occurs at the CNS, which triggers the release of corticosteroid and catecholamine hormones, and initiates the secondary responses including metabolic, haematological and osmoregulatory changes [6]. However, tissues or organs responsible for the sense of body temperature fluctuations and the thermoregulation in fish remain largely unknown and further efforts are needed to characterize signaling pathways and master genes involved in these processes.

In summary, this study identified multiple biological processes, intracellular signaling pathways and key genes that are potentially involved in the regulation of cold- and heat-stress acclimation in zebrafish.

## Materials and Methods

### Ethics Statement

The animal protocol for this study was approved by the Animal Care and Use Committee of Hubei Province in China and by the Institutional Animal Care and Use Committee of Institute of Hydrobiology (Approval ID: Keshuizhuan 0829).

### Chemicals

Most of chemicals with the highest purity available were obtained from China National Medicines Corporation LTD. Trypsin, BSA and HEPES were purchased from Amresco. Formaldehyde and tricane were obtained from Sigma.

### Temperature Stress Exposure

Zebrafish (*Danio rerio*) embryos were obtained as previously described [49]. The embryos were incubated at 28°C in 30% Danieau’s solution (19.3 mM NaCl, 0.23 mM KCl, 0.13 mM MgSO_4_·7H_2_O, 0.2 mM Ca(NO_3_)_2_, 1.67 mM Hepes at pH 7.2). Embryos at 48 hpf were randomly selected and maintained in 60 mm plastic petri dishes (60 larvae/dish) containing 6 ml embryo medium. For temperature stress exposure, larvae at 96 hpf (maintained at 28°C from fertilization) were transferred immediately into dishes containing pre-conditioned medium at 16 (cold), 28 (control) or 34°C (heat) and incubated in biochemical incubators under corresponding temperatures for 2 and 48 h, respectively. After exposed to temperature stress, samples were collected at 98 (treated for 2 h) and 144 hpf (treated for 48 h), respectively. Larvae at 96 hpf were used to perform the experiment because larvae at this stage need not to be fed and are less sensitive to temperature stress than the earlier stage embryos. The temperature and exposure time were referenced from previous studies [22] and determined by pre-experiments to ensure the occurrence of stress responses and reduce the mortality. To avoid the influence of light exposure on gene expression, embryos were kept in dark throughout the experiment. After temperature stress exposure for 48 h, zebrafish larvae were anesthetized with 0.016% tricaine and images were taken under a stereomicroscope from Zeiss with a color CCD camera.

### Alcian Blue Staining and Fish Measurement

The alcian blue staining of cartilages in zebrafish larvae was perform as previously described [50]. Stained preparations were mounted in 70% glycerol and photographed as described above. The standard lengths (SL) and eye diameter (ED) of live larvae and the distance from the inner border of Meckel’s cartilage to the anterior end of ceratohyal (DMC) and the ceratohyal length (CL) of stained larvae were measured with the AxioVision software (Zeiss). Each parameter was measured from 40–60 fish.

### Biochemical Analysis

To characterize the effects of temperature stress on body composition, biochemical parameters including wet mass, dry mass, protein, lipid and glycogen were analyzed. Each assay was performed three times independently and 5–10 biological replicates were included in each experiment. The mass of zebrafish larvae was determined by addition of pooled larvae into pre-weighted 2 mL Eppendorf tubes. The water was sucked out and the tubes were weighted again by a microbalance from Mettler. Then, the samples were dried at 60? for 24 h and weighted again. 80 individuals were used in each assay. The wet/dry mass was obtained through dividing the difference between wet/dry weight and tube weight by the number of larvae.

To determine the protein content, individual larvae were lysed in 50 µL of 0.5 M NaOH by two freeze-thaw cycles between −80 and 4°C. 5 µL of lysate was used to determine protein concentration with microplate assays using the DC protein assay kit from Bio-rad. The absorbance at 750 nm was determined with SpectraMax M5 microplate reader. The calibration curve was generated by using BSA as a standard.

Lipid content analysis was modified from Cheng et al. [51]. Briefly, 20 larvae were homogenized with chloroform/methanol (2∶1) in 1.5 mL tubes and centrifuged at 10000 rpm for 5 min. The supernatant was collected and evaporated in boiling water bath. Then 100 µL of concentrated sulfuric acid was added and heated in boiling water bath for 20 min. After kept on ice for 2 min, 150 µL of vanillin-phosphoric acid reagent (0.2 mg vanillin per ml 17% phosphoric acid) was added for color development and incubated for 10 min. The absorbance at 540 nm was determined with the microplate reader. The calibration curve was generated by using the commercial corn oil as a standard.

The glycogen analysis was modified from Templeton [52]. Briefly, 20 larvae were pooled in 1.5 mL tubes and homogenized in 200 µL of 30% KOH. The sample was heated in boiling water bath for 10 min. 120 µL of 2% Na2SO4 and 800 µL absolute ethanol were added to precipitate the glycogen. After spinning at 1500 g for 10 min, the supernatant was discarded and the pellet was washed with 1 ml of 66% ethanol. The pellet was dissolved in 200 µL water and 200 µL anthrone reagent (0.2% anthrone in 77% sulfuric acid) was then added to 50 µL sample or glucose standard. The sample was then kept in boiling water bath for 15 min to develop the color. After cooling at room temperature for 15 min, the absorbance at 625 nm was determined with the microplate reader.

### Total RNA Extraction

At desired time points, larvae were anesthetized by placing the culture dish on ice. 60 individuals cultured in the same dish were pooled and subjected to RNA extraction. Total RNA was extracted with TRIZOL Reagent from Invitrogen following the manufacturer’s instructions. Total RNA contents were determined by measuring the absorbance at 260 nm. The quality of RNA samples was assessed by agarose gel electrophoresis and ultraviolet spectrophotometry.

### RNA Labeling and Microarray

One-color microarray analysis for gene expression variation in zebrafish larvae was performed by ShanghaiBio Corporation (SBC) using the Agilent Zebrafish Oligo Microarray (V2) (4×44 K). Three biological replicates for each treatment at desired time points were independently collected and a total of 18 microarray assays were conducted. Each RNA sample used for microarray hybridization was extracted from 60 pooled larvae exposed to temperature stress as described above. The RNA integrity was confirmed by the check of RNA integrity number (RIN) with Agilent Bioanalyzer 2100. Qualified RNA samples were purified with the RNeasy mini kit (QIAGEN) and RNase-Free DNase Set (QIAGEN). Total RNA was amplified with the Low RNA Input Linear Amplification kit (Agilent technologies) and labeled with the 5-(3-aminoallyl)-UTP (Ambion) and Cy3 NHS ester (GE healthcare Biosciences) according to the manufacturer’s instructions. Labeled cRNA was purified with the RNeasy mini kit (QIAGEN). Each slide was hybridized with 1.65 µg Cy3-labeled cRNA using the Gene Expression Hybridization Kit (Agilent technologies). After hybridization for 17 h, slides were washed with the stabilization and drying solution in the Gene Expression Wash Buffer Kit (Agilent technologies). Slides were scanned using the Agilent Microarray Scanner and Feature Extraction software 10.7 with default settings. The raw data were normalized using quantile algorithm with Gene Spring Software 11.0 (Agilent technologies) and log2-transformed before further analysis. The raw and normalized data were stored in the ArrayExpress database (Accession NO. E-MTAB-983).

### Data Analysis of Microarrays

Probe sets with significant hybridization signal in temperature stress-exposed samples and corresponding controls were submitted to subsequent analysis. PCA was performed with Arraytrack [53] to elucidate the overall patterns of gene expression in larvae exposed to different temperature.

The SAM algorithm from Mev (Multiple array viewer) version 4.8 [54] was used to identify differentially expressed genes between two treatments. Developmental processes-associated genes were identified by the comparison of genes expressed at 144 hpf (48 h after exposure) with those at 98 hpf (2 h after exposure). Temperature stress-regulated genes were identified by the comparison of genes expressed in samples exposed to 16 or 34°C with those in corresponding controls maintained at 28°C. Only probes called “present” in more than 2 of the 3 replicates from both groups were input. A two-class unpaired grouping was selected and all permutations were used. The S0 parameter was calculated using the default method. Fold change ≥1.8 and FDR (false discover rate) <0.05 were set up as the threshold to identify the differentially expressed probes between two groups.

The design of microarrays used in this study is based on the Zv7 version of zebrafish genomic database, so 60 nt sequences of differentially expressed probes without annotated genes were blasted against the Refseq database of zebrafish at NCBI (http://www.ncbi.nlm.nih.gov/). Hit sequences containing alignment length ≥58 and mismatch ≤1 were regarded as exact matches. The accession numbers of matched sequences were converted to Entrez gene ID by g:Profiler [55]. Venn diagrams representing the numbers of differentially expressed genes were generated by ArrayTrack [56].

Up- or down-regulated genes at each time point were submitted to GO enrichment analysis using the GOEAST web based software (http://omicslab.genetics.ac.cn/GOEAST/index.php) [57] and the Agilent zebrafish (V2) gene expression microarray was used as reference. KEGG pathway enrichment analysis was performed with ClueGO [58], a plugin for Cytoscape [59]. Two-sided hypergeometric method was used for statistical test and the p values were corrected with the Benjamini-Hochberg method. Heatmaps describing enriched GO terms and pathways were produced using Gitools [60].

Hierarchical clustering using a Euclidean distance metric was performed to cluster the expression data of genes with Cluster 3.0 software. Heatmaps demonstrating the gene expression data were created by the Java TreeView software [61].

### Quantitative Real-time PCR

To validate the data of microarray, the expression of 15 genes, including *hmgb1*, *cirbp*, *hspb1*, *hsp47*, *nr1d4a*, *nr1d4b*, *nr5a5*, *her8a*, *per3*, *brf2*, *per2*, *nr0b2b*, *nr1d1*, *nr1d2a* and *per1b*, was determined by MIQE (Minimum information for publication of quantitative real-time PCR experiments)-compliant qPCR analysis of three independent biological replicates. The selection of genes for validation was based on future interests, up- and down-regulation, and previously reported responses to temperature stress.

First-strand cDNA for each sample was synthesized from 4 µg of total RNA using random hexamer primer with the RevertAid™ First Strand cDNA Synthesis Kit from Fermentas. The PCR primers were designed using Primer Premier 5.0 software. qPCR was performed in a MiQ Cycler from BioRad. The amplification was carried out in a volume of 20 µL containing 10 µL of 2 × SYBER Green Real Time PCR Master mix from TOYOBO, 2 pmol of each primer and 5 µL of 10×diluted cDNA templates. All reactions were carried out in triplicates. The qPCR conditions were as follows: 40 cycles of 10 s at 95°C and 30 s at 60°C, followed by the melting curve: 26 cycles of 30 s with an increase of 1°C between each cycle from 70°C to 95°C. The reaction specificity was confirmed by the observation of a single peak at the expected Tm on the melting curve. The amplification cycle displaying the first significant increase of the fluorescence signal was defined as threshold cycle and used for quantification (Cq).

Before qPCR analysis, the standard curve of each primer pair was generated by the regression of Cq values and a series of 10-fold cDNA dilutions from 96 hpf zebrafish larvae (Figure S1). The amplification efficiency of primers was calculated from the slope of corresponding standard curve. The sequences, Tm value and amplification efficiency of primers, the accession number, gene name of target sequences and the length of amplicons were listed in Table S1.

To identify the most stable internal reference, genes including *rpl7a*, *tnnt3b*, *tpma*, *tpt1* and *bactin* were selected as candidates according to their expression detected by microarray. The expression of these genes in all of the samples was also determined by qPCR. The expression data were analyzed using NormFinder [33] to identify the most stable gene and gene combination. The microarray and qPCR expression of candidate reference genes and the result of NormFinder analysis were displayed in Table S2. The geometric average of the Cq values and amplification efficiency of *tpma* and *tnnt3b* were used to calculate the relative expression of genes using the Q-Gene software [62].

### Statistical Analysis

SPSS 15.0 software for Windows was used for statistical analysis. One-way analysis of variance (ANOVA) followed by a Duncan’s post-hoc test (for equal variance) or Dunnett’s T3 test (for unequal variance) was performed to analyze the significant difference (*p*<0.05) in the measurable parameters and biochemical compositions of larvae exposed to different temperature. The correlation between the data of microarray and qPCR was analyzed by the Spearman’s rho test.

## Supporting Information

Figure S1 **Standard curves for qPCR primer pairs.**
(TIF)

Figure S2 **Rank-rank analysis heat map graph.**
(TIF)

Figure S3 **GO terms enriched in genes up-regulated by cold exposure for 2 h.**
(PDF)

Figure S4 **GO terms enriched in genes up-regulated by heat exposure for 2 h.**
(PDF)

Table S1 **Information for primers used in qPCR analysis.**
(XLS)

Table S2 **Selection of internal reference genes for qPCR analysis.**
(XLS)

Table S3 **Comparison between microarray and qPCR data.**
(DOC)

Table S4 **Expression of developmental processes-associated genes.**
(XLS)

Table S5 **Expression of temperature-regulated genes.**
(XLS)

Table S6 **Official symbols of differentially expressed genes.**
(XLS)

Table S7 **Results of GO enrichment analysis.**
(XLS)
