# Supplementary figures and images for: Transcriptomic Characterization of Temperature Stress Responses in Larval Zebrafish
Source: PLoS One. 2012 May 30;7(5):e37209. doi: 10.1371/journal.pone.0037209 (PMC3364249; doi:10.1371/journal.pone.0037209)

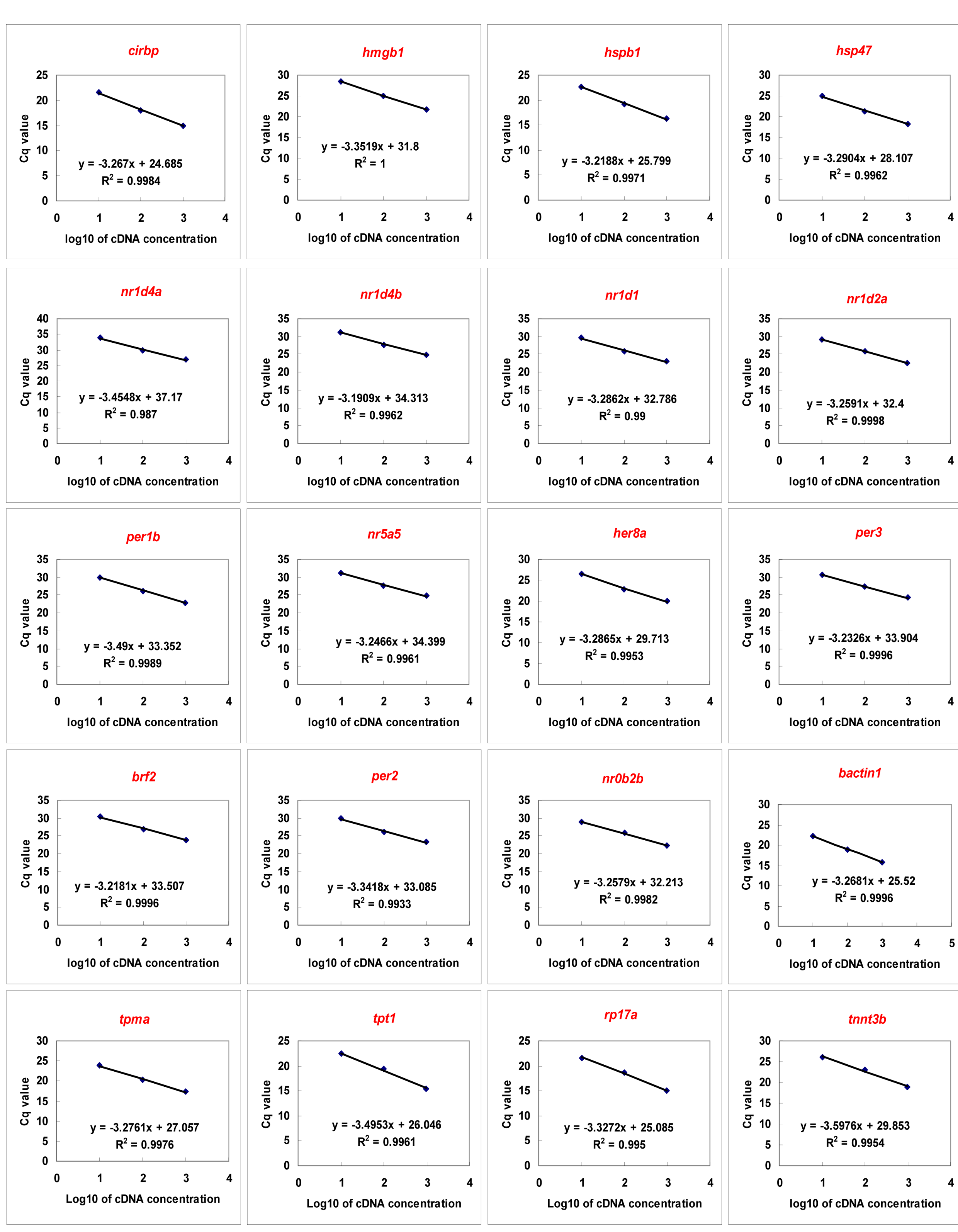

Supplement: Figure S1 — Standard curves for qPCR primer pairs. (TIF) [file pone.0037209.s001.tif]

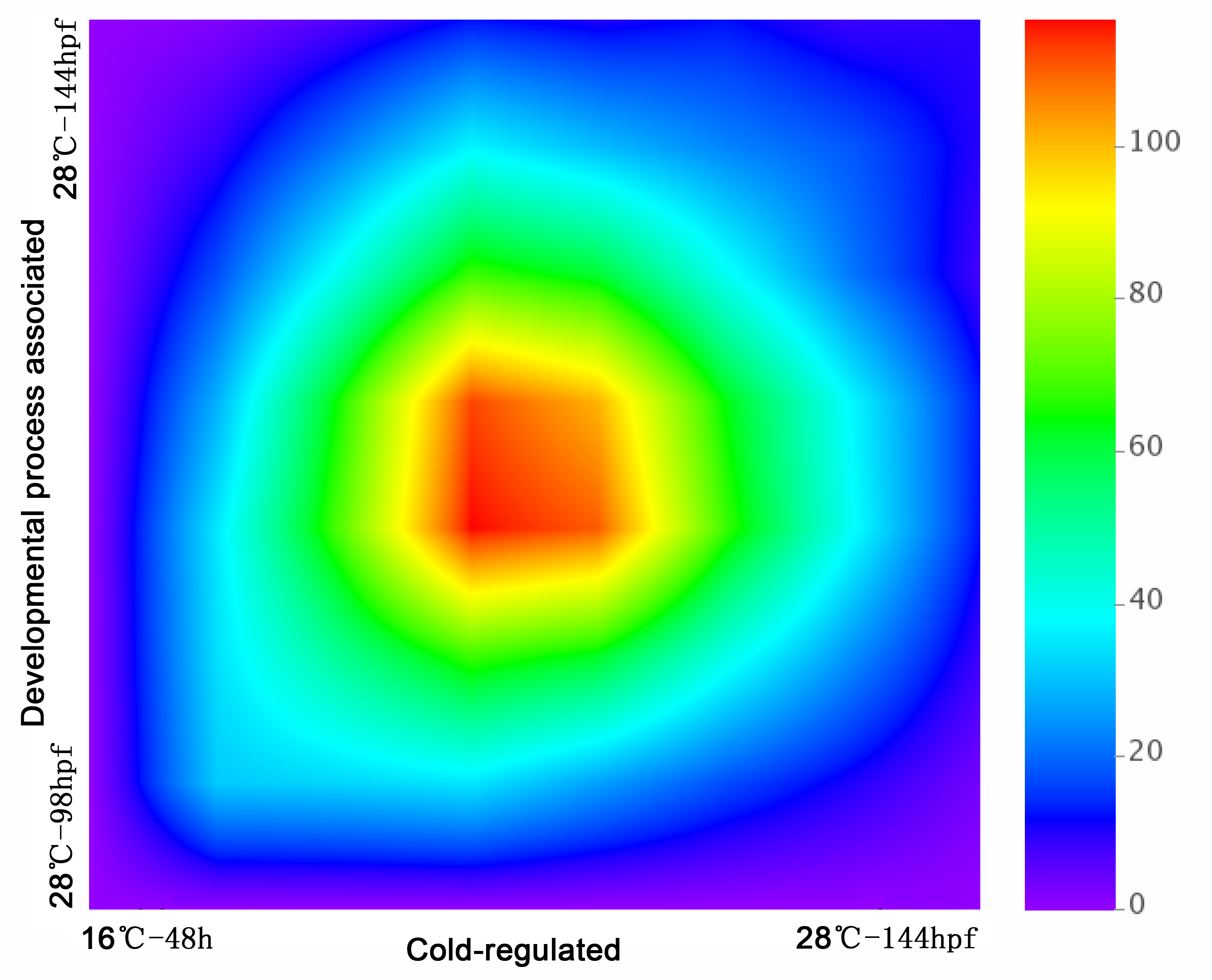

Supplement: Figure S2 — Rank-rank analysis heat map graph. (TIF) [file pone.0037209.s002.tif]

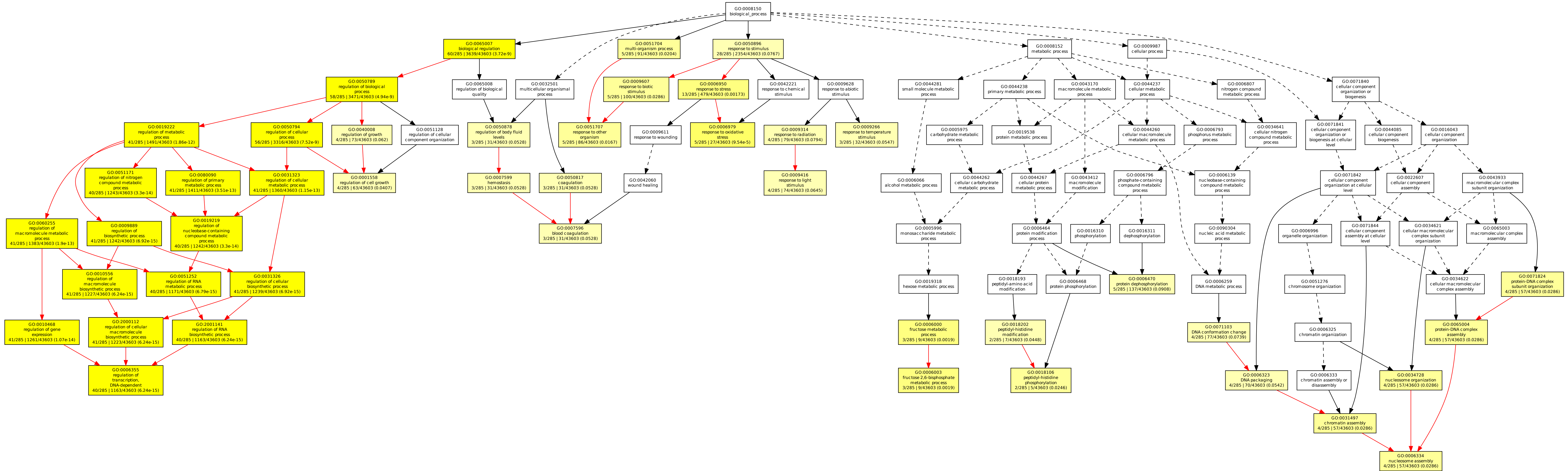

Supplement: Figure S3 — GO terms enriched in genes up-regulated by cold exposure for 2 h. (PDF) [file pone.0037209.s003.pdf]

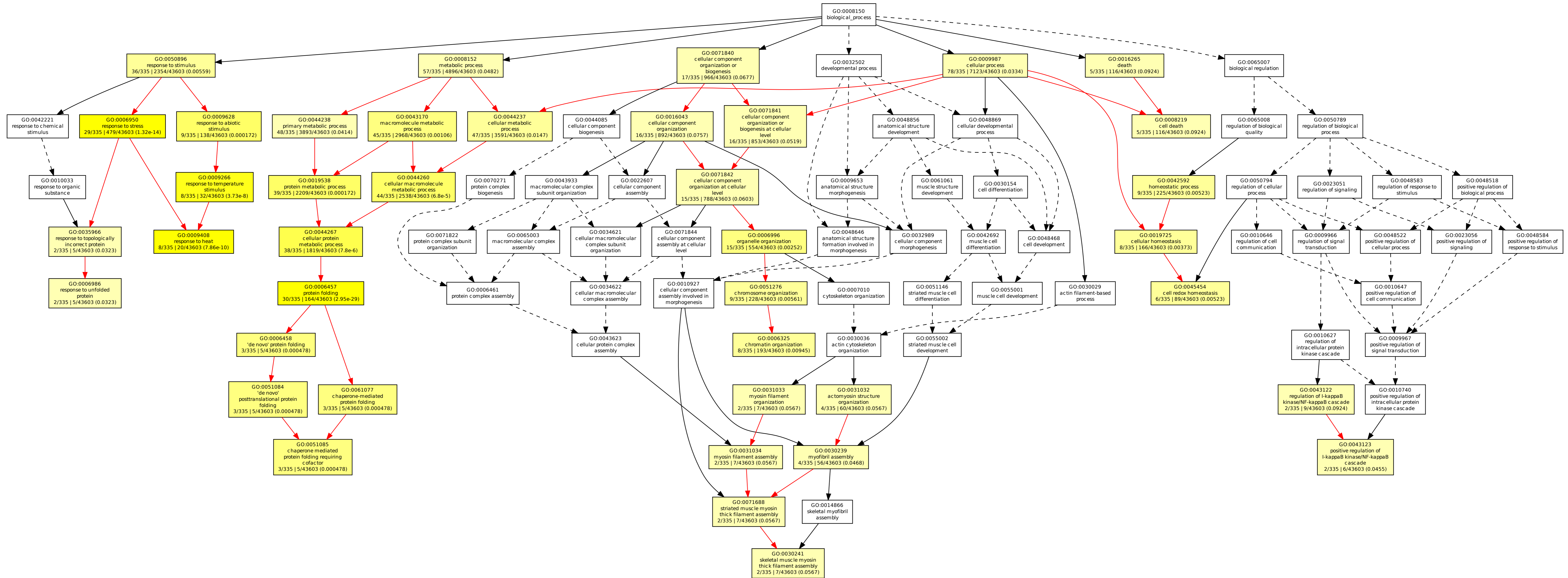

Supplement: Figure S4 — GO terms enriched in genes up-regulated by heat exposure for 2 h. (PDF) [file pone.0037209.s004.pdf]
