# Supplementary material for: Transcriptomic Characterization of Temperature Stress Responses in Larval Zebrafish
Source: PLoS One. 2012 May 30;7(5):e37209. doi: 10.1371/journal.pone.0037209 (PMC3364249; doi:10.1371/journal.pone.0037209)
Supplement: Table S3 — Comparison between microarray and qPCR data. (DOC) [file pone.0037209.s007.doc]

**Table S3. Comparison between microarray and qPCR data**

| **Gene names** | **Sample names** | **Microarray** | **qPCR** |
| --- | --- | --- | --- |
| *cirbp* | 16℃-2h | 1.09±0.06 | 1.09±0.20 |
|  | 16℃-48h | 2.56±0.08 | 1.96±0.15 |
| *hmgb1* | 16℃-2h | 1.09±0.03 | 1.07±0.27 |
|  | 16℃-48h | 2.54±0.14 | 1.97±0.38 |
| *hspb1* | 34℃-2h | 6.30±2.26 | 4.55±1.06 |
|  | 34℃-48h | 3.03±0.69 | 2.19±0.81 |
| *hspb47* | 34℃-2h | 13.66±2.57 | 14.75±0.91 |
|  | 34℃-48h | 18.52±7.62 | 7.59±1.00 |
| *nr5a5* | 16℃-48h | -6.83±2.44 | -25.95±5.46 |
| *her8a* | 16℃-48h | -2.35±0.16 | -10.72±6.35 |
| *per3* | 16℃-48h | -3.54±1.74 | -5.61±1.35 |
| *brf2* | 16℃-48h | 3.77±0.82 | 5.65±0.62 |
| *per2* | 16℃-48h | 4.50±0.51 | 8.04±3.09 |
| *nr0b2b* | 16℃-48h | 4.44±1.27 | 5.04±2.77 |
| *nr1d4a* | 16℃-2h | 9.30±1.94 | 6.78±0.69 |
| *nr1d4b* | 16℃-2h | 9.88±1.22 | 7.71±0.48 |
| *nr1d1* | 16℃-2h | 5.81±1.15 | 7.56±1.36 |
| *nr1d2a* | 16℃-2h | 5.30±1.06 | 4.51±1.33 |
| *per1b* | 16℃-2h | 4.36±0.64 | 4.26±0.89 |

Fold change was calculated by dividing gene expression values in temperature treated samples with those in corresponding controls maintained at 28℃. Values are given as mean ± standard deviation of three independent experiments.
